# Huddling Human Beings

**Published:** 1875-02

**Authors:** 


HUDDLING HUMAN BEINGS.—It would seem that not only all New York but the entire country rides in the horse-cars of this city. Statistics show that 140,500,000 passengers rode in our street-cars in 1874. This is equal to three times the population of the United States. The statement hints at a tale of misery which will never be printed. It tells of crowded conveyances, reeking with filth, odorous with emenations from unclean bodies, and prolific with contagion and infection. It speaks of small-pox, diptheria, and all manner of diseases carried from street to street and from house to house, by these ever-moving charnel-houses. It is fearful to contemplate the disease which has been engendered in these cars, and communicated to others. It is worse in winter than in summer, because the windows are open in summer, while in winter the ventilation is necessarily very imperfect. The inference that sufficient air enters around the loose windows and at the rear door when open, is erroneous. If the cars were twenty times as large they would not contain an adequate supply of air for the number of passengers now conveyed. Many lives would be saved which are now sacrificed, if all public conveyances in New York were to cease running, thus compelling most of the inhabitants to walk to and from their houses. But there are multitudes to whom a walk of even ten blocks would seem to be a fearful task. This class must ride of course. They have no idea of freedom, yet they would scorn to be accounted slaves, as they are. They are slaves to corsets, tight boots, indolence, and the fear that they will be laughed at if they manifest independence by walking.

So we must put up with the disease and dirt of the street-cars, until the day of "rapid transit" dawns, which, if it does not save us from the danger of infection, will at least abbreviate the period of daily contact with the infected.

